# Pharmacogenetics and Pharmacokinetics of Moxifloxacin in MDR-TB Patients in Indonesia: Analysis for *ABCB1* and *SLCO1B1*

**DOI:** 10.3390/antibiotics14020204

**Published:** 2025-02-16

**Authors:** Nurul Annisa, Nadiya N. Afifah, Prayudi Santoso, Vycke Yunivita, Lindsey H. M. te Brake, Rob E. Aarnoutse, Melisa I. Barliana, Rovina Ruslami

**Affiliations:** 1 Department of Biological Pharmacy, Faculty of Pharmacy, Universitas Padjadjaran, Jl. Ir. Soekarno Km. 21, Jatinangor 45363, West Java, Indonesia; nurul19008@mail.unpad.ac.id (N.A.); nadiya12001@mail.unpad.ac.id (N.N.A.); 2 Division of Clinical and Community Pharmacy, Faculty of Pharmacy, Universitas Mulawarman, Jl. Kuaro Gunung Kelua, Samarinda 75119, East Kalimantan, Indonesia; 3 Department of Pharmacy, Faculty of Health Sciences, Universitas Esa Unggul, Jl. Arjuna Utara, Kebun Jeruk, Jakarta 11510, Jakarta, Indonesia; 4 Division of Pulmonary and Critical Care, Faculty of Medicine, Department of Internal Medicine, Universitas Padjadjaran, Hasan Sadikin General Hospital, Jl. Prof. Eyckman, Bandung 40162, West Java, Indonesia; prayudi@unpad.ac.id; 5 Research Center for Care and Control of Infectious Disease, Universitas Padjadjaran, Jl. Prof. Eyckman, Bandung 40162, West Java, Indonesia; v.yunivita@unpad.ac.id (V.Y.); rovina.ruslami@unpad.ac.id (R.R.); 6 Division of Pharmacology and Therapy, Department of Biomedical Sciences, Faculty of Medicine, Universitas Padjadjaran, Jl. Ir. Soekarno Km. 21, Jatinangor 45363, West Java, Indonesia; 7 Department of Pharmacy, Radboud Institute for Medical Innovation, Radboud University Medical Center, Geert Grooteplein Zuid 10, 6525 GA Nijmegen, The Netherlands; lindsey.tebrake@radboudumc.nl (L.H.M.t.B.); rob.aarnoutse@radboudumc.nl (R.E.A.); 8 Center of Excellence for Pharmaceutical Care Innovation, Universitas Padjadjaran, Jl. Ir. Soekarno Km. 21, Jatinangor 45363, West Java, Indonesia

**Keywords:** pharmacogenetics, pharmacokinetics, AUC_0–24_, C_max_, moxifloxacin, *ABCB1* rs2032582, *SLCO1B1* rs4149015, MDR-TB

## Abstract

**Background/Objectives**: Studies show that SNPs in *ABCB1* rs2032582 and *SLCO1B1* rs4149015 affect the PK profile of moxifloxacin, a key drug for MDR-TB. This study aimed to assess the genotype frequencies of *ABCB1* rs2032582 and *SLCO1B1* rs4149015; describe moxifloxacin AUC_0–24_ and C_max_; and evaluate the association between genotype variations and moxifloxacin AUC_0–24_ and C_max_, corrected for the effect of other determinants in MDR-TB patients in Indonesia. **Methods**: The genotypes were identified using DNA sequencing. Plasma samples for PK analysis were collected at either two or four timepoints post-dose, at steady state. AUC_0–24_ values were assessed with a limited sampling formula. A multivariate linear regression analysis identified the determinants for moxifloxacin AUC_0–24_ and C_max_. **Results**: We recruited 204 MDR-TB patients for PG analysis, with 80 providing PK samples. The majority of the *ABCB1* and *SLCO1B1* genotypes were wildtype (GG), 41.7% and 93.6%, respectively. The geometric mean AUC_0–24_ for moxifloxacin was 78.6 mg·h/L and that for C_max_ was 6.1 mg/L. No statistically significant difference in exposure to moxifloxacin could be shown between the genotypes. Sex, age, and dose in mg/kg/body weight were significant determinants of the AUC_0–24_ of moxifloxacin. **Conclusions**: The major genotype of *ABCB1* rs2032582 and *SLCO1B1* rs4149015 was wildtype, and the exposure to moxifloxacin was high but not related to the studied genotype in an Indonesian population.

## 1. Introduction

Multidrug-resistant tuberculosis (MDR-TB) poses a significant challenge, necessitating the optimization of treatment strategies [1]. The fluoroquinolone antibiotics moxifloxacin and levofloxacin are crucial for treating MDR-TB and are designated as group A MDR-TB drugs. In so-called shorter (9–11 months) multi-drug MDR-TB treatment regimens, moxifloxacin or levofloxacin are part of the initial phase (4–6 months) as well as the continuation phase (5 months) of treatment [2]. Most recently, an even shorter (6 month) regimen was introduced that comprises bedaquiline, pretomanid, linezolid, and moxifloxacin (BPaLM) [3].

Moxifloxacin exerts its bactericidal effect on *Mycobacterium tuberculosis* by inhibiting topoisomerase II (deoxyribonucleic acid (DNA)-gyrase) and topoisomerase IV, essential enzymes for bacterial DNA processes [4,5]. The efficacy of moxifloxacin relies on achieving a target area under the plasma concentration for the 0–24 h (AUC_0–24_)/MIC ratio, in which AUC represents the total exposure to a drug in the plasma [6]. The total exposure to a drug can be influenced by various factors, including variations in genes involved in pharmacokinetic (PK) mechanisms. These variations include single-nucleotide polymorphisms (SNPs) in transporter genes. Indeed, ATP-binding cassette (ABC) proteins and solute carrier (SLC) transporters exhibit polymorphisms that impact the AUC_0–24_ and C_max_ of moxifloxacin. Moxifloxacin is a substrate for p-glycoprotein (P-gp), a transporter encoded by the ATP-binding cassette subfamily B member 1 (*ABCB1)* gene. Studies have indicated that a polymorphism in *ABCB1* can lead to a decrease in the AUC_0–24_ and an increase in the time to reach the maximum concentration (C_max_) of moxifloxacin (t_max_) [7]. Furthermore, research involving pulmonary TB patients from Africa and the USA revealed that a variant g.—11187G>A in solute carrier organic anion transporter family member 1B1 (*SLCO1B1*) rs4149015 was associated with an increase in both the AUC_0–24_ and C_max_ of moxifloxacin [8]. However, no studies on the association between pharmacogenetics (PG) and the PK of moxifloxacin have been conducted in an Indonesian population, particularly in treating MDR-TB, although Indonesia ranks number two for tuberculosis prevalence [1].

Therefore, the objectives of this study were (1) to describe the *ABCB1* rs2032582 and *SLCO1B1* rs4149015 genotype distribution in Indonesian MDR-TB patients; (2) to describe the AUC_0–24_ and C_max_ of moxifloxacin in Indonesian MDR-TB patients; and (3) to assess the association between *ABCB1* rs2032582 and *SLCO1B1* rs4149015 SNPs and moxifloxacin AUC_0–24_ and C_max_, corrected for the effect of other predictors.

## 2. Results

**Figure 1:**
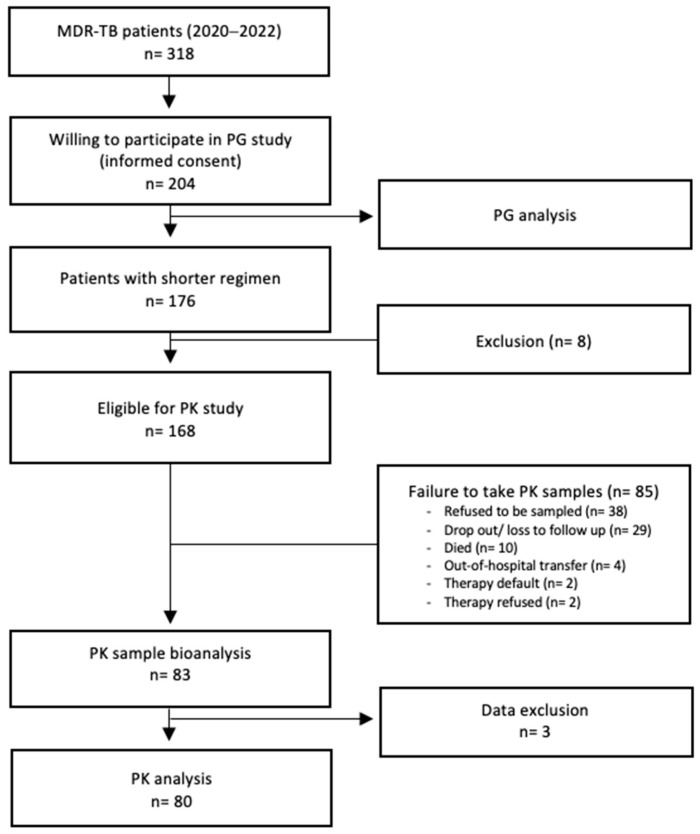
Patient tree.

From June 2020 to May 2022, 204 patients treated at the Hasan Sadikin Hospital in Bandung were recruited to participate in the PG study. Of these, 168 were eligible for inclusion in the PK study (Figure 1).

Of the 168 eligible individuals, 83 were enrolled in the PK substudy. Three subjects were eliminated due to outlier data. The patients in the PK substudy were categorized into two groups, based on the daily dosage of moxifloxacin received (400 mg or 600 mg) based on their body weight.

The patient characteristics, data on drug regimens, and laboratory results of the patients are summarized in Table 1a,b. The majority of patients were male with a median age of 39–40 years and predominantly an underweight status. 

The data show that patients, on average, displayed normal laboratory parameter values, including liver and renal parameters.

The distribution of the genotype frequencies (n = 204) is summarized in Table 2. For *ABCB1* rs2032582, the majority showed a wildtype GG (41.7%), while, for *SLCO1B1* rs4149015, the majority genotype was wildtype GG (93.6%).

In patients with PK data (n = 80, Table 3), the geometric mean of the moxifloxacin AUC_0–24_ was 78.6 mg·h/L, while, for C_max_, it was 6.2 mg/L. 

Sex demonstrated significant associations with AUC_0–24_ and C_max_. Female patients showed higher exposure to moxifloxacin, either AUC_0–24_ (110.0 vs. 60.5 mg·h/L, *p* < 0.001) or C_max_ (7.7 vs. 5.2 mg/L, *p* < 0.001) than male patients, while patients with a higher age showed higher AUC_0–24_ values (*p* = 0.049; see Table 3). In addition, a significant difference was observed in moxifloxacin C_max_ between patients on all-oral regimens and those on a regimen with a kanamycin injection (6.5 vs. 4.4 mg/L, *p* = 0.030). Both the AUC_0–24_ and the C_max_ of moxifloxacin were also correlated with dose per body weight (r = 0.292, *p* = 0.004 and r = 0.361, *p* = 0.001).

**Figure 2:**
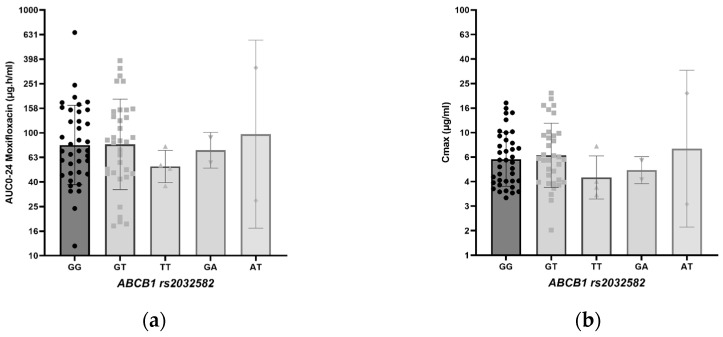
(**a**) Boxplot *ABCB1* rs2032582 genotype with moxifloxacin geometric mean AUC_0–24_; (**b**) boxplot *ABCB1* rs2032582 genotype with moxifloxacin geometric mean C_max_.

The effect of *ABCB1* rs2032582 and *SLCO1B1* rs4149015 genotype variation on moxifloxacin AUC_0–24_ and C_max_ is shown in Table 4 and Figure 2. No statistically significant difference in exposure to moxifloxacin could be shown between the genotypes, although the geometric mean AUC_0–24_ and C_max_ were lower in the TT genotype in *ABCB1* rs2032582 than that in the other genotypes, while the geometric mean AUC_0–24_ (128.2 h.mg/L) and C_max_ (8.4 mg/L) were higher in the GA genotype of *SLCO1B1* rs4149015 (Table 4).

Figure 2 depicts a boxplot comparing the *ABCB1* rs2032582 genotypes (GG, GT, TT, GA, and AT) with the moxifloxacin geometric mean AUC_0–24_ and C_max_ values.

**Figure 3:**
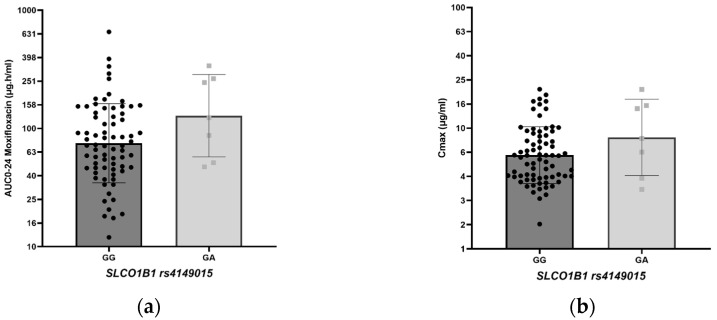
(**a**) Boxplot *SLCO1B1* rs4149015 genotype with moxifloxacin geometric mean AUC_0–24_; (**b**) boxplot *SLCO1B1* rs4149015 genotype with moxifloxacin geometric mean C_max_.

Figure 3 depicts a boxplot comparing the *SLCO1B1* rs4149015 genotypes (GG and GA) with the moxifloxacin geometric mean AUC_0–24_ and C_max_ values.

The subsequent multiple linear regression analysis revealed that sex and dose in mg/kg predicted the AUC_0–24_ and C_max_ of moxifloxacin, whereas age also affected the AUC_0–24_ (Table 5).

## 3. Discussion

This is the first study on a PG analysis concerning the PK of moxifloxacin within the Indonesian population. The first objective of this study was to assess the genotypes of *ABCB1* rs2032582 and *SLCO1B1* rs4149015 in MDR-TB patients in Indonesia (PG study). Secondly, the moxifloxacin AUC_0–24_ and C_max_ were described in eligible participants (PK study). The third objective was to evaluate the association between genotype variants and the AUC_0–24_ and C_max_ of moxifloxacin.

Within the dataset of the PG substudy, the most frequently observed *ABCB1* rs2032582 genotypes were GG, GT, TT, GA, and AT, with the major genotype being the wildtype GG. These findings align with genotype patterns identified in the Chinese Han population, characterized by GG, GT, and TT [9]. Additionally, the genotype variations observed in our study were consistent with the results of another study conducted in the Polish population [10]. Meanwhile, other studies within a South African population showed a predominantly CC genotype [11]. As for *SLCO1B1* rs4149015, genotype GG was shown to be the major genotype, which is also the wildtype, with GA as a variant. This is consistent with another study conducted in white and black populations, which revealed the same genotype distribution and frequency [8].

When only considering the moxifloxacin dose of 400 mg once daily, the exposure to moxifloxacin in MDR-TB patients in this study was higher than that in drug-sensitive pulmonary TB patients [12] or TB meningitis patients [13] from the same Indonesian setting. The AUC_0–24_ values were 71.4, 48.2, and 33.6, while those for C_max_ were 5.7, 4.7, and 7.4, respectively. The AUC_0–24_ of moxifloxacin in the MDR-TB patients in this study was also higher than that of the TB patients in another heterogeneous population (78.6 vs. 33.6 mg·h/L) [14]. A higher exposure to 400 mg of moxifloxacin in this study may partly be explained by the (absent) effect of rifampicin on the metabolism of moxifloxacin [12]. Furthermore, considering both the 400 and 600 mg moxifloxacin doses, it should be considered that over 60% of patients in our study had a low body weight, and the average moxifloxacin dose received was high at 10.3 mg/kg of body weight (Table 1b).

This study found no association between genotype variations in *ABCB1* and *SLCO1B1* and the AUC_0–24_ and C_max_ of moxifloxacin. Still, the analysis on *SLCO1B1* rs4149015 revealed a trend that patients with the GA genotype exhibited a higher moxifloxacin AUC_0–24_ and C_max_ than those with the GG genotype (Table 4). This result is consistent with a previous study conducted in Africa and the United States. In the study of *SLCO1B1* g.11187, the variant genotype AG had a median moxifloxacin AUC_0–24_ that was 46% higher and a median C_max_ that was 30% higher than that for the GG genotype (wildtype) [8].

In this study, a multiple linear regression analysis revealed that sex, age, and dose/kg body weight were significant predictors for moxifloxacin AUC_0–24_, while sex and dose per body weight were predictors for C_max_. One possible reason for the sex-related differences in the PK might be due to variations in the regulation of drug metabolism through endogenous hormonal influences. It may involve a combination of genetics and physiological factors [15]. The differential tissue expression of P-gp between the genders has been reported as a significant contributor to gender differences in both the PK and pharmacodynamic (PD) response observed between the genders for many of its drug substrates [16]. As for organic anion transporter polypeptide (OATP) expression (encoded by the *SLCO1B1* gene) in age and gender, these variations can influence drug disposition and efficacy and may be the basis for drug interactions, especially in children and the elderly [17]. Another study noted a higher exposure to moxifloxacin in elderly females than that in elderly men. However, no significant differences were found when normalized to body weight, and no serious adverse effects were associated [18]. Age or gender had no effect on the bioavailability of levofloxacin, another fluoroquinolone, as reported by another study [19].

In addition to *ABCB1* and *SLCO1B1*, other genes such as uridine 5′-diphosphate-glucuronosyltransferase family 1 member A1 (*UGT1A1*) and member A9 (*UGT1A9*) also influence the PK parameters of moxifloxacin, but they were not analyzed in this study [7,11,20], which is a notable limitation. Another limitation is the relatively small sample size in our PG and PK studies. A larger sample size is essential for a more comprehensive understanding of the relationship between genotype variations and moxifloxacin PK in Indonesian MDR-TB patients.

## 4. Materials and Methods

### 4.1. Patients and Study Design

This study was part of the MDR-TB cohort study conducted between 2020 and 2022 at the Hasan Sadikin Hospital, Bandung, a referral hospital for the West Java province in Indonesia. The diagnosis of MDR-TB was based on a rapid molecular test (Xpert^®^ MTB-RIF Assay G4) for the identification of rifampicin resistance in *M. tuberculosis* from sputum samples. The MDR-TB therapeutic regimen to be given by the clinician was based on the line probe assay (LPA) results. LPA is a DNA strip-based rapid test designed to determine the drug resistance profile by detecting the most common mutations associated with resistance to first- and second-line anti-TB agents, as well as specific *M. tuberculosis* wildtype DNA sequences.

All the MDR-TB patients were sampled for the PG study. Adult (>18 years old) MDR-TB patients who received a short-course (9–11 months) regimen containing moxifloxacin were recruited for the PK study. Pregnant and breastfeeding females were excluded from this PK study. This study was approved by the Research Ethical Committee of Universitas Padjadjaran (No. 643/UN6.KEP/EC/2020). Eligible patients were asked for written informed consent.

### 4.2. PG and PK Study

Samples for the PG study were taken from the leftovers during routine laboratory examinations at baseline by taking the patient’s whole blood. The DNA-isolation process involved the use of the Gene Elute™ Mammalian Genomic DNA Miniprep kit (Sigma Aldrich, Merck, Darmstadt, Germany, catalog number G1N10). Subsequently, the isolated DNA was quantified with a spectrophotometer and stored at −20 °C until further analysis. Gene sequences were obtained from the National Center for Biotechnology Information (https://www.ncbi.nlm.nih.gov) (accessed on 16 September 2021). For *ABCB1* rs2032582, the specific primers used were as follows: forward primer for *ABCB1* rs2032582: 5′-GAGCATAGTAAGCAGTAGGGAGT-3′ and reverse primer: 5′-GCAGGCTATAGGTTCCAGGC-3′; forward primer for *SLCO1B1*: 5′-GGCCTTGGGTCTACATTTCTCA-3′ and reverse primer: 5′-AGTACAGACCCTTCTCTCACA-3′ (Macrogen, Singapore, Singapore). A polymerase chain reaction (PCR) was carried out using the GoTaq™ Green Master Mix (Promega, Madison, WI, USA). The PCR conditions included denaturation at 95 °C for 2 min, annealing at 61 °C for *ABCB1* rs2032582 and 59 °C for *SLCO1B1* rs4149015, extension at 72 °C for 1 min, and a final extension at 72 °C for 10 min, with a total of 30 cycles. The PCR products underwent 2% agarose gel electrophoresis and were visualized at a 312 nm wavelength using a Biometra instrument. The expected amplicon size was 298 bp for *ABCB1* rs2032582 and 365 bp for *SLCO1B1* rs4149015. Genotyping was further confirmed through a DNA sequencing/capillary electrophoresis method.

PK sampling. PK sampling for moxifloxacin was conducted at the patient’s scheduled follow-up, typically one month after initiating drug treatment, when a steady state for moxifloxacin can be expected. Serial blood samples (2 mL each) were collected to assess the plasma concentrations of moxifloxacin at specific time points (at 0, 2, 4, and 6 h post-dose). If this was not possible (because patients could not stay longer at the clinic), the blood samples were collected at 0 and 2 h post-dose. Blood sampling was performed using a single insertion of a venous catheter. The blood samples were centrifuged at 3000 rpm for 15 min, and plasma was separated and stored at −80 °C within 30 min after sampling.

Bioanalysis of PK samples. The plasma concentrations of moxifloxacin were measured using high-performance liquid chromatography (HPLC) with UV detection. The mobile phase consisted of triethanolamine (TEA) (0.4%) in Milli-Q^®^ water (pH ±3) and acetonitrile 100% with an eluent ratio of 75:25%. A Sunfire™ Column C-18 (4.6 × 100 mm, 5 µL: Waters™: Ireland) served as the stationary phase, and detection was achieved using a Waters detector 2998 photodiode array (PDA) at 296 nm. The calibration range was 0.20–10.2 mg/L for human plasma. The intra-day and inter-day imprecision, expressed as the coefficient of variation (%CV), were shown to be lower than 7.2% and 5.0%, respectively, at all the concentrations tested, and the accuracies were between 95.5% and 103.4%.

PK data analysis. The AUC_0–24_ of moxifloxacin was calculated using the limited sampling formula developed by Magis et al. [14]. The formula to calculate AUC_0–24_ was [AUC_0–24_ = −4.35 + (3.97 × C2) − (6.49 × C4) + (20.05 × C6)] for subjects who had concentration data at 0, 2, 4, and 6 h after drug administration [14]. For subjects who had concentration data at 0 and 2 h post-dose, moxifloxacin exposures were calculated using the formula: AUC_0–24_ = [5.056 + (31.687 × C0) + (4.413 × C2)]. The C_max_ value was the highest measured concentration in a patient.

Statistical analysis. The patient characteristics in the PG and PK studies as well as the genotype distributions in the PG study were presented descriptively. Moxifloxacin AUC_0–24_ and C_max_ values obtained in the PK study were presented as geometric mean and minimum–maximum values. Differences in the AUC_0–24_ and C_max_ values between genotypes or between patient subgroups based on age, sex, body mass index (BMI), comorbidity, intake of drugs with or without food, moxifloxacin dose, and drug regimen were assessed using an unpaired t-test for 2 groups or a one-way ANOVA in the case of more than 2 groups, after log-transformation of the PK data. The moxifloxacin AUC_0–24_ and C_max_ were correlated with dose in mg/kg by using Spearman rank correlation.

After these univariate analyses, possible predictors with *p* < 0.20 were included in a multiple linear regression to identify moxifloxacin AUC_0–24_ and C_max_ predictors. Statistical significance was achieved with *p* < 0.05. All the statistical analyses were performed in SPSS software version 22. The graphs were generated using GraphPad Prism software (version 9.2.0; GraphPad Software, San Diego, CA, USA).

## 5. Conclusions

In conclusion, our observation found that the major genotype of *ABCB1* rs2032582 and *SLCO1B1* rs4149015 was GG. The average AUC_0–24_ and C_max_ values of moxifloxacin were high at 78.6 mg·h/L and 6.1 mg/L, respectively. There was no association between genotype frequencies with AUC_0–24_ and C_max_. Multivariate analysis revealed that sex, age, and dose per body weight were significant determinants for AUC_0–24_, while sex and dose per body weight were predictors for C_max_.

## Figures and Tables

**Table 1:** (**a**). Patient characteristics. (**b**). Laboratory results and drug regimens of patients in the PK study.

(a)		
Variable	PG Study (N = 204)	PK Study (N = 80)
Sex		
Male	150 (58.8)	45 (56.3)
Age (years), median (min–max)	39 (18–81)	40 (18–66)
Body weight (kg), median (min–max)	46.0 (29–84)	44.5 (34–73)
BMI		
Overweight	9 (4.4)	5 (6.3)
Normal	84 (41.2)	24 (30.0)
Underweight	111 (54.4)	51 (63.7)
Comorbidity		
Diabetes mellitus	44 (21.6)	14 (17.5)
Hypertension	14 (6.9)	5 (6.3)
**(b)**		
**Variable**		**N = 80**
Drug regimen		
Injection containing		11 (13.8)
All oral		69 (86.2)
Moxifloxacin daily dose		
400 mg		41 (51.2)
600 mg		39 (48.8)
Dose/body weight (mg/kg), median (min–max)		10.3 (8.2–14.0)
Time of taking medicine before sampling (hours), median (min–max)		23 (11–27)
Number of days taking medicine, median (min–max)		31 (14–126)
Taking with meal		
Yes		33 (41.3)
Laboratory results		
Albumin (g/dL), median (min–max)		3.4 (2.2–4.6)
Abnormal		35 (43.7)
Normal (3.5–5 g/dL)		45 (56.3)
ALT (U/L), median (min–max)		25 (11–721)
Low level		4 (5.3)
Normal level (14–59 U/L)		63 (82.9)
High level		9 (11.8)
eGFR (mL/min), median (min–max)		110 (49–330)
eGFR criteria, n (%)		n = 76
Abnormal		1 (1.3)
Normal (>60 mL/min)		75 (98.7)

(a) Data are presented in n (%) or as otherwise stated. BMI: body mass index, MDR-TB: multidrug-resistant tuberculosis. (b) Data are presented as n (%) unless otherwise stated. ALT: alanine aminotransferase, eGFR: estimated glomerular filtration rate. Drug regimen with injections: moxifloxacin—isoniazid high dose—kanamycin (injection)—ethionamide—pyrazinamide—ethambutol. All-oral regimen: moxifloxacin—isoniazid high dose—bedaquiline—ethionamide—pyrazinamide—ethambutol.

**Table 2:** Genotype frequencies of *ABCB1* rs2032582 and *SLCO1B1* rs4149015.

Variable	PG Study N = 204	PK Study N = 80
*ABCB1* rs2032582, n (%)		
Genotype		
GG	85 (41.7)	38 (47.5)
GT	77 (37.7)	34 (42.5)
TT	26 (12.7)	4 (5.0)
GA	11 (5.4)	2 (2.5)
AT	5 (2.5)	2 (2.5)
*SLCO1B1* rs4149015, n (%)		
Genotype		
GG	191 (93.6)	73 (91.3)
GA	13 (6.4)	7 (8.7)

**Table 3:** Moxifloxacin AUC_0–24_ and C_max_ with determinants.

Variable	n	AUC_0–24_ (mg·h/L)	*p*-Value	C_max_ (mg/L)	*p*-Value
All	80	78.6 (12.0–656.8)	-	6.1 (1.6–21.0)	-
Sex					
Male	45	60.5 (12.0–387.6)	<0.001 *	5.2 (41.6–21.0)	<0.001 *
Female	35	110.0 (37.0–656.8)		7.7 (3.2–20.9)	
Age (year)					
18–34	31	63.9 (12.0–339.4)	0.049 *	5.8 (1.6–20.9)	0.359
35–49	30	78.0 (18.9–335.2)		5.9 (3.1–18.9)	
50–66	19	111.5 (45.0–656.8)		7.2 (3.3–21.0)	
BMI					
Overweight	5	84.1 (45.0–335.21)	0.660	6.0 (3.3–18.9)	0.815
Normal	24	88.1 (24.9–656.8)		6.5 (3.1–17.5)	
Underweight	51	74.0 (12.0–387.6)		6.0 (1.6–21.0)	
Intake with meal					
Yes	47	87.3 (12.0–387.6)	0.154	6.7 (1.6–21.0)	0.131
No	33	67.7 (17.4–656.8)		5.5 (2.8–17.5)	
Comorbidity					
DM status					
Yes	14	113.2 (45.0–263.6)	0.054	6.8 (3.3–15.4)	0.457
No	66	72.7 (12.0–656.8)		6.0 (1.6–21.0)	
Hypertension					
Yes	5	87.0 (46.4–656.8)	0.765	5.2 (3.2–17.5)	0.502
No	75	78.0 (12.0–387.6)		6.2 (1.6–21.0)	
Moxifloxacin dose					
400 mg	41	71.4 (17.4–339.4)	0.262	5.7 (2.6–20.9)	0.274
600 mg	39	86.9 (12.0–656.8)		6.6 (1.6–21.0)	
Drug regimen					
Injection containing	11	67.7 (37.0–108.7)	0.501	4.4 (3.1–6.5)	0.030 *
All oral	69	80.5 (12.0–656.8)		6.5 (1.6–21.0)	
Dose per body weight **	80	R = 0.3	0.004 *	R = 0.361	0.001 *

AUC_0–24_: area under the plasma concentration versus time curve from 0 to 24 h (total exposure), C_max_: peak plasma concentration, BMI: body mass index, DM: diabetes mellitus. Analyses used the unpaired *t*-test (2 groups) and one-way ANOVA test (>2 groups); * significant *p* < 0.05; ** analysis used Spearman’s rank correlation.

**Table 4:** The effect of *ABCB1* rs2032582 and *SLCO1B1* rs4149015 gene polymorphisms on the moxifloxacin AUC_0–24_ and C_max_.

Variable	n	AUC_0–24_ (mg·h/L)	*p*-Value	C_max_ (mg/L)	*p*-Value
		Geometric Mean (Min–Max)		Geometric Mean (Min–Max)	
*ABCB1* rs2032582					
GG	38	79.5 (12.0–656.8)	0.883	6.1 (2.9–17.5)	0.651
GT	34	80.5 (17.4–387.6)		6.5 (1.6–21.0)	
TT	4	53.3 (37.0–77.6)		4.3 (3.1–7.7)	
GA	2	72.2 (57.0–91.4)		4.9 (4.1–5.9)	
AT	2	97.6 (28.0–339.4)		7.4 (2.6–20.9)	
*SLCO1B1* rs4149015					
GG	73	75.0 (12.0–656.8)	0.083	6.0 (1.6–21.0)	0.064
GA	7	128.2 (47.2–339.4)		8.4 (3.1–20.9)	

AUC_0–24_: area under the plasma concentration versus time curve from 0 to 24 h (total exposure), C_max_: peak plasma concentration (AUC_0–24_ and C_max_ were log-transformed in analyses). Analyses used the unpaired *t*-test (2 groups), one-way ANOVA test (>2 groups), min: minimum, max: maximum.

**Table 5:** Predictors of moxifloxacin AUC_0–24_ and C_max_.

Variable	Unstandardized Coefficients		Standardized Coefficients	*p*-Value
	B	Std. Error	Beta	
Moxifloxacin AUC_0–24_				
(Constant)	0.927	0.274		0.001 *
Female sex	0.221	0.071	0.324	0.003 *
Age	0.007	0.003	0.278	0.014 *
Dose per body weight (mg/kg)	0.052	0.024	0.221	0.033 *
Dependent variable: Log AUC_0–24_ moxifloxacin, R^2^ = 0.282 Moxifloxacin C_max_				
(Constant)	−0.034	0.235		0.887
Female sex	0.125	0.052	0.256	0.020 *
Dose per body weight (mg/kg)	0.040	0.018	0.236	0.027 *
Dependent variable: Log C_max_, R^2^ = 0.180				

Analyses used were multiple linear regression on log-transformed AUC_0–24_ and C_max_ values. * Significant *p* < 0.05.

## Data Availability

Dataset available on request from the authors.

## Abbreviations

MDR-TB	Multidrug-resistant tuberculosis
PK	Pharmacokinetics
PG	Pharmacogenetics
BPaLM	Bedaquiline, pretomanid, linezolid, and moxifloxacin
DNA	Deoxyribonucleic acid
SNPs	Single-nucleotide polymorphisms
ABC	ATP-binding cassette
SLC	Solute carrier
P-gp	p-Glycoprotein
C_max_	The maximum concentration
T_max_	The time to reach the maximum concentration
AUC_0–24_	Area under the plasma concentration
*ABCB1*	*ATP-Binding Cassette Subfamily B Member 1*
*SLCO1B1*	*Solute Carrier Organic Anion Transporter Family Member 1B1*
*UGT1A1*	*Uridine 5′-diphosphate-glucuronosyltransferase family 1 member A1*
LPA	Line probe assay
PCR	Polymerase chain reaction
HPLC	High-performance liquid chromatography
TEA	Triethanolamine
PDA	Photodiode array
CV	Coefficient of variation
BMI	Body mass index
ALT	Alanine aminotransferase
eGFR	Estimated glomerular filtration rate
PD	Pharmacodynamic
OATPs	Organic anion transporter polypeptides
